# A genomic region associated with protection against severe COVID-19 is inherited from Neandertals

**DOI:** 10.1073/pnas.2026309118

**Published:** 2021-02-15

**Authors:** Hugo Zeberg, Svante Pääbo

**Affiliations:** ^a^Department of Evolutionary Genetics, Max Planck Institute for Evolutionary Anthropology, D-04103 Leipzig, Germany;; ^b^Department of Neuroscience, Karolinska Institutet, SE-17177 Stockholm, Sweden;; ^c^Human Evolutionary Genomics Unit, Okinawa Institute of Science and Technology, Okinawa 904-0495, Japan

**Keywords:** Neandertals, COVID-19, OAS1, SARS-CoV-2

## Abstract

It was recently shown that the major genetic risk factor associated with becoming severely ill with COVID-19 when infected by severe acute respiratory syndrome coronavirus 2 (SARS-CoV-2) is inherited from Neandertals. New, larger genetic association studies now allow additional genetic risk factors to be discovered. Using data from the Genetics of Mortality in Critical Care (GenOMICC) consortium, we show that a haplotype at a region on chromosome 12 associated with requiring intensive care when infected with the virus is inherited from Neandertals. This region encodes proteins that activate enzymes that are important during infections with RNA viruses. In contrast to the previously described Neandertal haplotype that increases the risk for severe COVID-19, this Neandertal haplotype is protective against severe disease. It also differs from the risk haplotype in that it has a more moderate effect and occurs at substantial frequencies in all regions of the world outside Africa. Among ancient human genomes in western Eurasia, the frequency of the protective Neandertal haplotype may have increased between 20,000 and 10,000 y ago and again during the past 1,000 y.

## Significance

We show that a haplotype on chromosome 12, which is associated with a ∼22% reduction in relative risk of becoming severely ill with COVID-19 when infected by SARS-CoV-2, is inherited from Neandertals. This haplotype is present at substantial frequencies in all regions of the world outside Africa. The genomic region where this haplotype occurs encodes proteins that are important during infections with RNA viruses.

Neandertals evolved in western Eurasia about half a million years ago and subsequently lived largely separated from the ancestors of modern humans in Africa (1), although limited gene flow from Africa is likely to have occurred (2–5). Neandertals as well as Denisovans, their Asian sister group, then became extinct about 40,000 y ago (6). However, they continue to have a biological impact on human physiology today through genetic contributions to modern human populations that occurred during the last tens of thousands of years of their existence (e.g., refs. 7–10).

Some of these contributions may reflect adaptations to environments outside Africa where Neandertals lived over several hundred thousands of years (11). During this time, they are likely to have adapted to infectious diseases, which are known to be strong selective factors that may, at least partly, have differed between sub-Saharan Africa and Eurasia (12). Indeed, several genetic variants contributed by archaic hominins to modern humans have been shown to affect genes involved in immunity (e.g., refs. 7, 8, 13, 14). In particular, variants at several loci containing genes involved in innate immunity come from Neandertals and Denisovans (15), for example, toll-like receptor gene variants which decrease the susceptibility to *Helicobacter pylori* infections and the risk for allergies (16). Furthermore, proteins interacting with RNA viruses have been shown to be encoded by DNA regions introgressed from Neandertals more often than expected (17), and RNA viruses might have driven many adaptive events in humans (18).

Recently, it was shown that a haplotype in a region on chromosome 3 is associated with becoming critically ill upon infection with the novel severe acute respiratory coronavirus 2 (SARS-CoV-2) (19) and was contributed to modern humans by Neandertals (20). Each copy of this haplotype approximately doubles the risk of its carriers requiring intensive care when infected by SARS-CoV-2. It reaches carrier frequencies of up to ∼65% in South Asia and ∼16% in Europe, whereas it is almost absent in East Asia. Thus, although this haplotype is detrimental for its carriers during the current pandemic, it may have been beneficial in earlier times in South Asia (21), perhaps by conferring protection against other pathogens, whereas it may have been eliminated in East Asia by negative selection.

A new study from the Genetic of Mortality in Critical Care (GenOMICC) consortium, which includes 2,244 critically ill COVID-19 patients and controls (22), recently became available. In addition to the risk locus on chromosome 3, it identifies seven loci with genome-wide significant effects located on chromosomes 6, 12, 19, and 21. Here, we show that, at one of these loci, a haplotype associated with reduced risk of becoming severely ill upon SARS-CoV-2 infection is derived from Neandertals.

## Results and Discussion

### A Neandertal Haplotype on Chromosome 12.

We investigated whether the index single-nucleotide polymorphisms (SNPs), that is, the SNPs with the strongest association (*Materials and Methods*), at the seven loci associated with risk of requiring intensive care upon SARS-CoV-2 infection on chromosomes 6, 12, 19, and 21 (22) harbor Neandertal-like alleles. To this end, we required that one of the alleles of the index SNPs should match all three high-quality Neandertals genomes, while being absent in the genomes of 108 African Yoruba individuals [*r*^2^ > 0.80; the 1000 Genomes Project (23)]. None of the index SNPs for the loci on chromosomes 6, 19, and 21 fulfilled these criteria, whereas the locus on chromosome 12 did.

**Fig. 1.**
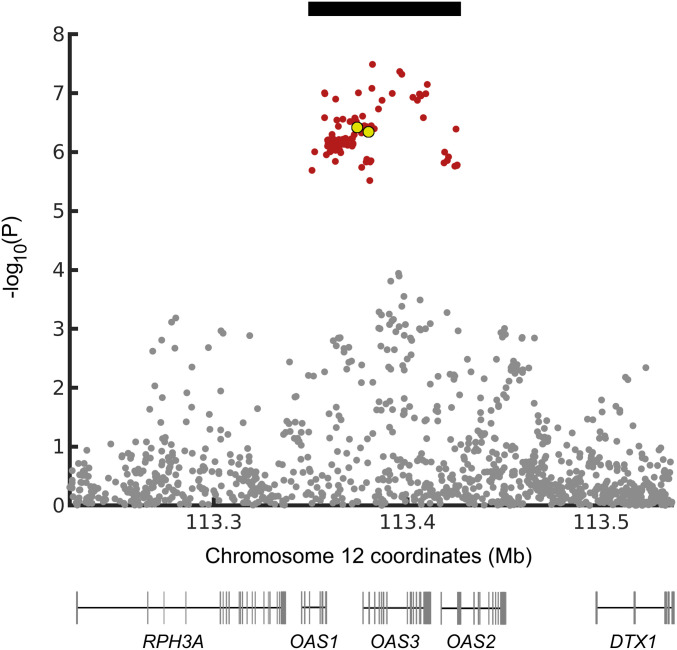
Genetic variants associated with COVID-19 hospitalization at the *OAS* locus. Variants marked in red have *P* values less than 1e-5. In Europeans, they are in LD with the index variant (*r*^2^ ≥ 0.8), forming a haplotype (black bar) with the genomic coordinates chr12: 113,350,796 to 113,425,679. *P* values are from the HGI (24), excluding the 23andMe data for which only sparse SNP data are available. The *x* axis gives *hg19* coordinates; genes in the region are indicated below. The three *OAS* genes are transcribed from left to right. Yellow dots indicate rs10735079 (right, the GenOMICC index SNP) and rs1156361 (left, typed by the Human Origins Array).

To further investigate this locus, we used data from the COVID-19 Host Genetics Initiative [HGI; round 4 (24)]. We find that the SNPs in the chromosome 12 locus associated with COVID-19 hospitalization (*P* < 1.0e-5; Fig. 1) are in linkage disequilibrium (LD) (*r*^2^ ≥ 0.8) in Europeans and form a haplotype of ∼75 kb (chr12: 113,350,796 to 113,425,679; *hg19*). LD to the index SNP of the GenOMICC study is given in *SI Appendix*, Table S1. Haplotypes of this length carrying alleles absent in Yoruba but present in Neandertals are likely to have been introduced into the gene pool of modern humans due to interbreeding with Neandertals (25).

**Fig. 2.**
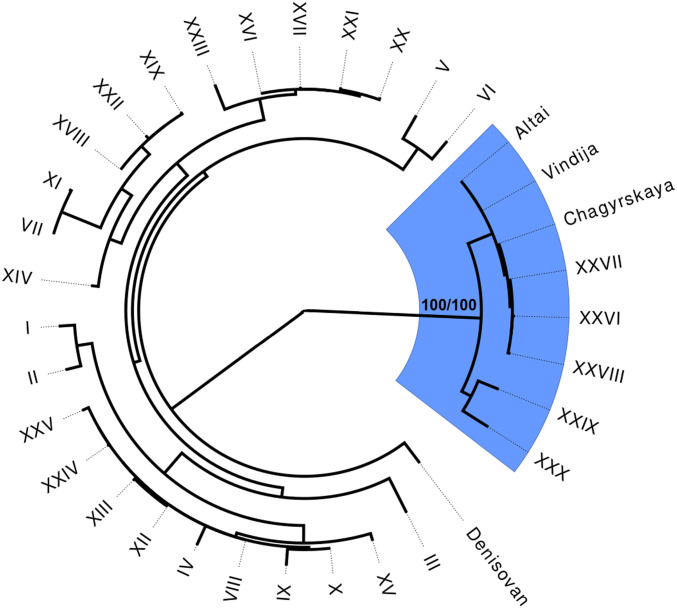
Phylogeny relating DNA sequences associated with COVID-19 severity on chromosome 12. Haplotypes from three Neandertal genomes, the Denisovan genome, and haplotypes seen more than 20 times in individuals in the 1000 Genomes Project are included. The colored area indicates haplotypes that carry the protective allele at rs1156361. The tree is rooted with the inferred ancestral sequence from Ensembl (46). Six heterozygous positions in the archaic genomes were excluded. Haplotypes XXIX and XXX are partially made up of Neandertal-like DNA sequences due to recombination events.

To test whether the 75-kb haplotype is the result of gene flow from Neandertals, we analyzed its relationship to present-day and archaic genomes. To do this, we used the haplotypes seen more than 10 times among the individuals in the 1000 Genomes Project (23) and the genome sequences of a ∼70,000-y-old Neandertal from Chagyrskaya Cave in southern Siberia (26), a ∼50,000-y-old Neandertal from Vindija Cave in Croatia (27), a ∼120,000-y-old Neandertal from Denisova Cave in southern Siberia (1), and a ∼80,000-y-old Denisovan individual from the same site (28). Fig. 2 shows a phylogenetic tree estimating the relationships among these haplotypes. Among the 64 modern human haplotypes, eight form a monophyletic group with the three Neandertal sequences.

Genomic segments with similarity to Neandertal genomes may either derive from common ancestors of the two groups that lived about half a million years ago or be contributed by Neandertals to modern humans by mixing between the two groups when they met less than 100,000 y ago (25). To test whether a segment of 75 kb may have survived in this region of the genome since the common ancestor of the groups without being broken down by recombination that affects chromosomes in each generation, we use a published equation (29), a generation time of 29 y (30), a regional recombination rate of 0.80 cM/Mb (31), and a split time between Neandertals and modern humans of 550,000 y (1) followed by interbreeding ∼50,000 y ago. Under these assumptions, in this region, segments of length 16.3 kb or longer are not expected to derive from the population ancestral to Neandertals and modern humans (*P* = 0.05), making it highly unlikely that a 75-kb haplotype does so (*P* = 8.2e-9). We thus conclude that the haplotype entered the human gene pool from Neandertals. In agreement with this, a previous study (32) has described gene flow from Neandertals in this genomic region.

### COVID-19 Protection and Geographic Distribution.

We find that the index variant of the protective haplotype in the GenOMICC study (rs10735079, *P* = 1.7e-8) matches all three Neandertal genomes available. The relative risk of needing intensive care is reduced by ∼22% per copy of the Neandertal haplotype (under the rare disease assumption, odds ratio [OR] = 0.78, 95% CI 0.71 to 0.85). As expected given the phylogeny (Fig. 2), almost all of the alleles cosegregating with the protective allele of the index SNP are found in the Neandertal genomes (34 of 35 called SNPs; see *SI Appendix*, Table S2, which, in contrast to Fig. 1, includes data contributed by 23andMe to HGI).

**Fig. 3.**
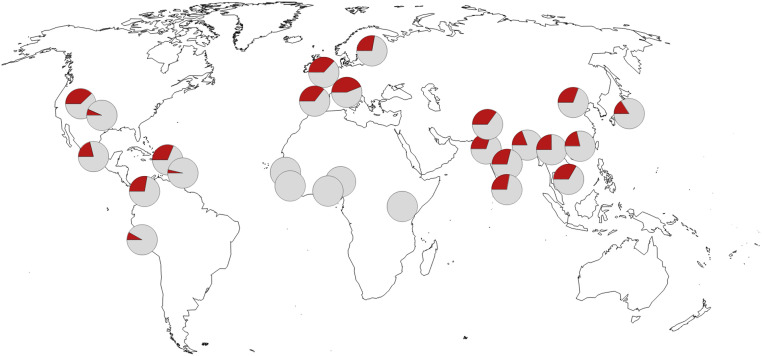
Geographic distribution of the allele indicative of the Neandertal haplotype protective against severe COVID-19. Pie charts indicate minor allele frequency in red at rs1156361. Frequency data are from the 1000 Genomes Project (23). Map source data are from OpenStreetMap.

Today, the haplotype is almost completely absent in African populations south of the Sahara but exists at frequencies of ∼25 to 30% in most populations in Eurasia (Fig. 3). In the Americas, it occurs in lower frequencies in some populations of African ancestry, presumably due to gene flow from populations of European or Native American ancestry (33).

### Putative Functional Variants.

The Neandertal haplotype protective against severe COVID-19 on chromosome 12 contains parts or all of the three genes *OAS1, OAS2*, and *OAS3*, which encode oligoadenylate synthetases. These enzymes are induced by interferons and activated by double-stranded RNA. They produce short-chain polyadenylates, which, in turn, activate ribonuclease *L*, an enzyme that degrades intracellular double-stranded RNA and activates other antiviral mechanisms in cells infected by viruses (reviewed by ref. 34).

To investigate which of these genes might be involved in protection against severe COVID-19, we plot the genomic location of the *OAS* genes below the *P* values for the SNPs associated with severe COVID-19 (Fig. 1). While the association (*P* < 1.0e-5) overlaps all three *OAS* genes, the SNPs with the most significant associations (*P* < 5.0e-8) are in *OAS3*. However, the high level of LD and stochasticity in the associations make any conclusion regarding causality based on *P* values tenuous.

Nevertheless, there are alleles on the Neandertal haplotype which stand out as potentially functionally important. One SNP (rs10774671) has been described as affecting a splice acceptor site in *OAS1* (35). The derived allele at this SNP, which is the most frequent allele in present-day humans, alters splicing of *OAS1* transcript such that several protein isoforms are produced instead of the ancestral isoform which is preserved in Neandertals (p46) (36). The latter, Neandertal-like isoform has higher enzymatic activity than the derived isoforms common in modern humans (37). Outside Africa, the ancestral allele is present only in the context of the Neandertal haplotype, whereas, in Africa, it exists independently of this haplotype, presumably as a genetic variant inherited from the common ancestors of modern humans and Neandertals that was lost in modern human populations that left Africa (35).

In addition to the splice acceptor site, the Neandertal haplotype contains a missense variant (rs2660) in *OAS1*, a missense variant (rs1859330) and two synonymous variants (rs1859329 and rs2285932) in *OAS3*, and a missense variant in *OAS2* (rs1293767). Three of these Neandertal-like variants are ancestral and occur in Africa (rs2660, rs1859330, and rs1859329), whereas two are derived in Neandertals (rs2285932 and rs1293767).

Several SNPs on the chromosome 12 haplotype have previously been studied with respect to their effects on other viral infections. The Neandertal-like splice acceptor variant has been associated with protection against West Nile Virus (rs10774671, OR = 0.63, 95% CI 0.5–0.83) (38), and the Neandertal-like haplotype has been associated with increased resistance to hepatitis C infections (39). Notably, the Neandertal missense variant in *OAS1* (rs2660) (or variants in LD with this variant) has been shown to be associated with moderate to strong protection against SARS-CoV [OR = 0.42, 95% CI: 0.20 to 0.89 (40)], although this study was limited in numbers of cases and controls. The SARS-CoV is closely related to SARS-CoV-2, emerged in 2003, and caused a mortality rate of ∼9% among infected individuals of all ages, and much higher rates of fatalities in older individuals (41). Finally, the Neandertal versions of the *OAS* genes are expressed differently in response to different viral infections in cells in tissue culture in terms of both expression levels and splice forms (35).

### Haplotype Frequencies across Time.

During the past few years, genome-wide data from thousands of prehistoric humans have been generated and compiled (42). This makes it possible to begin to directly gauge how frequencies of genetic variants have changed over time. Although this approach is still limited by the relatively small numbers of individuals and geographic regions for which data are available, we apply it here for the two Neandertal-derived haplotypes that affect the clinical outcomes upon infection with SARS-CoV-2.

To tag the Neandertal *OAS* haplotype on chromosome 12, we use an SNP (rs1156361) that carries a derived Neandertal-like allele, is associated with the index variant of the GenOMICC study (*r*^2^ = 0.99 in Eurasia), and is typed by the Affymetrix Human Origins array used to study the majority of ancient human genomes used here (42). Although this analysis is limited in that it tracks a single tag SNP, the fact that it is derived on the Neandertal lineage and in LD with the Neandertal haplotype makes this analysis feasible. We restrict the analysis to Eurasia and divide the data into five time windows that vary between 20,000 and 2,000 y in length, to balance the number of genomes available while still allowing potential differences in frequency to be discerned.

**Fig. 4.**
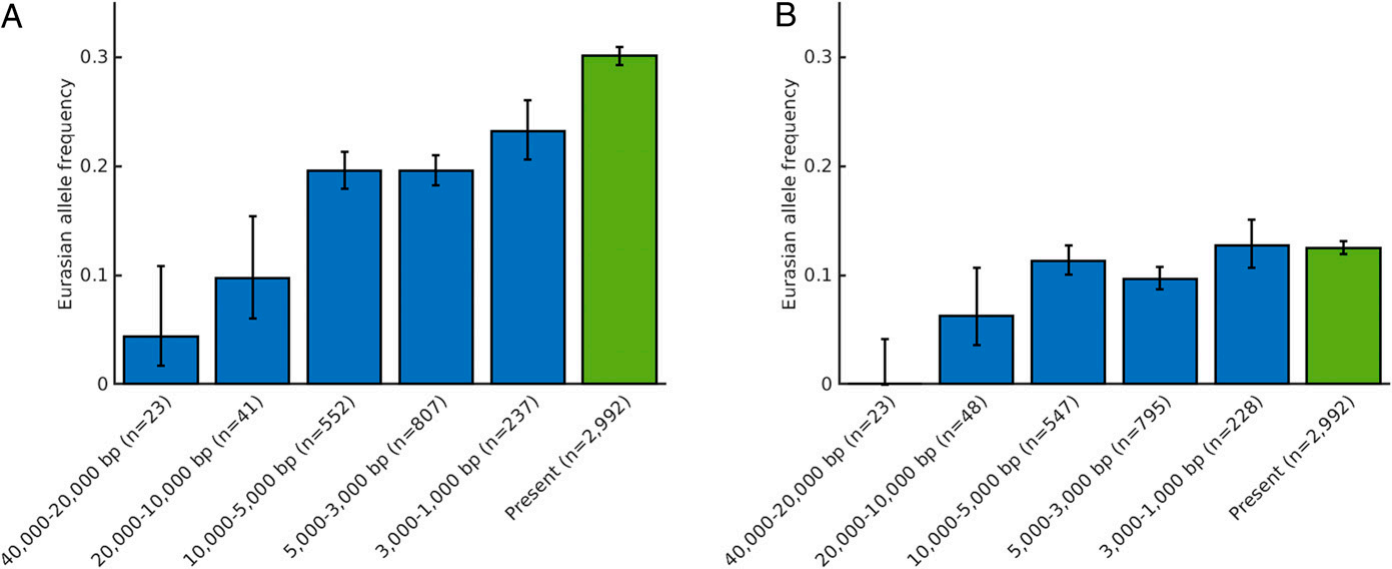
Frequencies across time of two Neandertal haplotypes associated with COVID-19 severity. Frequencies for rs1156361 at the *OAS* locus on chromosome 12 (*A*) and rs10490770 at the chromosome 3 locus (*B*). Error bars indicate SE (Wilson scores). Time periods are indicated in years before present (bp). Ancient data are from a compiled dataset (42), and present-day data are from the 1000 Genomes Project (23).

Fig. 4*A* shows that the Neandertal *OAS* haplotype seems to have occurred at frequencies below 10% prior to 20,000 y ago. Between 20,000 and 10,000 y ago, the allele frequency was in the order of 15%. Subsequently, it seems to have been present at frequencies at or slightly below 20% until 3,000 y to 1,000 y ago. Intriguingly, the current allele frequency in Eurasia is ∼30%, suggesting that the Neandertal *OAS* haplotype may have increased in frequency relatively recently.

To similarly estimate the frequency of the Neandertal risk haplotype on chromosome 3 (20), we use the SNP rs10490770 that fulfills the criteria applied above for the chromosome 12 haplotype (Fig. 4*B*). Prior to 20,000 y ago, we find no carrier of the risk haplotype among 16 genomes available. Among individuals who lived between 20,000 and 10,000 y ago and later, the haplotype is present in ∼10% until today, when it occurs at a frequency of ∼12.5%. Thus, similar to the *OAS* locus, the Neandertal chromosome 3 locus, the frequency seems to be lower in the period prior to 20,000 y ago than in the later periods. However, the data are still scarce, making this observation preliminary. In contrast to the *OAS* locus, there is no indication of any increase in the frequency of the Neandertal haplotype on chromosome 3 in historical times.

We caution that the prehistoric data available are heavily biased toward western Eurasia and are still sparse, particularly for older periods. However, additional data from ancient human remains are rapidly being generated, making us confident that it will soon be possible to identify loci that may have been the targets of positive and negative selection, by studying allele frequencies over time in certain geographical regions while correcting for migration events that caused genome-wide shifts in allele frequencies.

Despite theses caveats, it is interesting that the Neandertal-derived *OAS* locus has recently increased in frequency in Eurasia. This is compatible with previous work on the variation among present-day populations (32, 35, 43) suggesting that this locus has been positively selected. It is also compatible with Denisovans having contributed a version of this locus, which carries ancestral variants, for example, at the slice acceptor site (rs10774671), to people in Oceania, where it occurs at substantial frequencies today (44).

### Conclusions.

A Neandertal haplotype on chromosome 12 is protective for severe disease in the current SARS-CoV-2 pandemic. It is present in populations in Eurasia and the Americas at carrier frequencies that often reach and exceed 50%. The ancestral Neandertal *OAS* locus variants may thus have been advantageous to modern humans throughout Eurasia, perhaps due to one or many epidemics involving RNA viruses, especially given that the Neandertal haplotype has been found to be protective for at least three RNA viruses (West Nile virus, hepatitis C virus, SARS-CoV). Supporting this notion, simulations have demonstrated that the Neandertal *OAS* haplotype has been under positive selection in modern humans (35). Strikingly, the OAS1 protein encoded by the modern human *OAS* haplotype is of lower enzymatic activity than the one encoded by the Neandertal haplotype (37). This may have been advantageous at some point in Africa, because loss-of-function mutations of the *OAS1* locus have occurred numerous times among primates (45), suggesting that the maintenance of OAS1 activity is costly to an organism. One may speculate that, when modern humans encountered new RNA viruses outside Africa, the higher enzymatic activity of the ancestral variants that they acquired through genetic interactions with Neandertals may have been advantageous.

Intriguingly, there is evidence that the Neandertal-like *OAS* haplotype may have recently increased in frequency in Eurasia (Fig. 4*A*), suggesting that selection may have positively affected the Neandertal-derived *OAS* locus in the last millennium. Future studies of human remains from historical times will clarify whether, and when, this occurred.

## Materials and Methods

The index variants for the seven novel loci (rs9380142, rs143334143, rs3131294, rs10735079, rs74956615, rs2109069, and rs2236757) were obtained from GenOMICC (22). The regional summary statistics from the round 4 release of the metaanalysis carried out by the COVID-19 HGI (24) (https://covid19hg.org/results) was used to analyze the chromosome 12 locus (hospitalized vs. population controls, i.e., “B2” phenotype, using all ancestries but not including the 23andMe study, due to limited release of number of variants). LD was calculated using LDlink 4.1, and alleles were compared to the archaic genomes using tabix (HTSlib 1.10). The haplotype associated with protection against severe COVID-19 was investigated using phylogenetic software (PhyML 3.0), and the probability of observing a haplotype of a certain length or longer due to incomplete lineage sorting was calculated as described (29). The present-day haplotypes were constructed by including all variable positions in the region chr12: 113,350,796 to 113,425,679, excluding singletons. Haplotypes seen more than 10 times were included in the phylogenetic analysis. The inferred ancestral states at variable positions among present-day humans were taken from Ensembl. Genotypes of ancient genomes of modern humans were obtained from a compiled database (42). Maps displaying allele frequencies of different populations were made using Mathematica 11.0 (Wolfram Research, Inc.) and OpenStreetMap data.

## Supplementary Material

Supplementary File

## Data Availability

Previously published data were used for this work (COVID-19 HGI 1000 Genomes Project).

## References

[1] K. Prüfer., The complete genome sequence of a Neanderthal from the Altai Mountains. Nature 505, 43–49 (2014). 24352235 10.1038/nature12886 PMC4031459

[2] M. Kuhlwilm., Ancient gene flow from early modern humans into Eastern Neanderthals. Nature 530, 429–433 (2016). 26886800 10.1038/nature16544 PMC4933530

[3] M. Meyer., Nuclear DNA sequences from the Middle Pleistocene Sima de los Huesos hominins. Nature 531, 504–507 (2016). 26976447 10.1038/nature17405

[4] C. Posth., Deeply divergent archaic mitochondrial genome provides lower time boundary for African gene flow into Neanderthals. Nat. Commun. 8, 16046 (2017). 28675384 10.1038/ncomms16046 PMC5500885

[5] M. Petr., The evolutionary history of Neanderthal and Denisovan Y chromosomes. Science 369, 1653–1656 (2020). 32973032 10.1126/science.abb6460

[6] T. Higham., The timing and spatiotemporal patterning of Neanderthal disappearance. Nature 512, 306–309 (2014). 25143113 10.1038/nature13621

[7] C. N. Simonti., The phenotypic legacy of admixture between modern humans and Neandertals. Science 351, 737–741 (2016). 26912863 10.1126/science.aad2149 PMC4849557

[8] M. Dannemann, J. Kelso, The contribution of Neanderthals to phenotypic variation in modern humans. Am. J. Hum. Genet. 101, 578–589 (2017). 28985494 10.1016/j.ajhg.2017.09.010 PMC5630192

[9] H. Zeberg., A Neanderthal sodium channel increases pain sensitivity in present-day humans. Curr. Biol. 30, 3465–3469.e4 (2020). 32707058 10.1016/j.cub.2020.06.045

[10] H. Zeberg, J. Kelso, S. Pääbo, The Neandertal progesterone receptor. Mol. Biol. Evol. 37, 2655–2660 (2020). 32437543 10.1093/molbev/msaa119 PMC7475037

[11] F. Racimo, S. Sankararaman, R. Nielsen, E. Huerta-Sánchez, Evidence for archaic adaptive introgression in humans. Nat. Rev. Genet. 16, 359–371 (2015). 25963373 10.1038/nrg3936 PMC4478293

[12] E. K. Karlsson, D. P. Kwiatkowski, P. C. Sabeti, Natural selection and infectious disease in human populations. Nat. Rev. Genet. 15, 379–393 (2014). 24776769 10.1038/nrg3734 PMC4912034

[13] L. Abi-Rached., The shaping of modern human immune systems by multiregional admixture with archaic humans. Science 334, 89–94 (2011). 21868630 10.1126/science.1209202 PMC3677943

[14] H. Quach., Genetic adaptation and Neandertal admixture shaped the immune system of human populations. Cell 167, 643–656.e17 (2016). 27768888 10.1016/j.cell.2016.09.024 PMC5075285

[15] M. Deschamps., Genomic signatures of selective pressures and introgression from archaic hominins at human innate immunity genes. Am. J. Hum. Genet. 98, 5–21 (2016). 26748513 10.1016/j.ajhg.2015.11.014 PMC4716683

[16] M. Dannemann, A. M. Andrés, J. Kelso, Introgression of Neandertal- and Denisovan-like haplotypes contributes to adaptive variation in human toll-like receptors. Am. J. Hum. Genet. 98, 22–33 (2016). 26748514 10.1016/j.ajhg.2015.11.015 PMC4716682

[17] D. Enard, D. A. Petrov, Evidence that RNA viruses drove adaptive introgression between Neanderthals and modern humans. Cell 175, 360–371.e13 (2018). 30290142 10.1016/j.cell.2018.08.034 PMC6176737

[18] D. Enard, D. A. Petrov, Ancient RNA virus epidemics through the lens of recent adaptation in human genomes. Philos. Trans. R. Soc. Lond. B Biol. Sci. 375, 20190575 (2020). 33012231 10.1098/rstb.2019.0575 PMC7702803

[19] D. Ellinghaus., Genomewide association study of severe Covid-19 with respiratory failure. N. Engl. J. Med. 383, 1522–1534 (2020). 32558485 10.1056/NEJMoa2020283 PMC7315890

[20] H. Zeberg, S. Pääbo, The major genetic risk factor for severe COVID-19 is inherited from Neanderthals. Nature 587, 610–612 (2020). 32998156 10.1038/s41586-020-2818-3

[21] S. R. Browning, B. L. Browning, Y. Zhou, S. Tucci, J. M. Akey, Analysis of human sequence data reveals two pulses of archaic Denisovan admixture. Cell 173, 53–61.e9 (2018). 29551270 10.1016/j.cell.2018.02.031 PMC5866234

[22] E. Pairo-Castineira.; GenOMICC Investigators; ISARICC Investigators; COVID-19 Human Genetics Initiative; 23andMe Investigators; BRACOVID Investigators; Gen-COVID Investigators, Genetic mechanisms of critical illness in Covid-19. Nature, 10.1038/s41586-020-03065-y (2020).

[23] A. Auton.; 1000 Genomes Project Consortium, A global reference for human genetic variation. Nature 526, 68–74 (2015). 26432245 10.1038/nature15393 PMC4750478

[24] COVID-19 Host Genetics Initiative, The COVID-19 Host Genetics Initiative, a global initiative to elucidate the role of host genetic factors in susceptibility and severity of the SARS-CoV-2 virus pandemic. Eur. J. Hum. Genet. 28, 715–718 (2020). 32404885 10.1038/s41431-020-0636-6 PMC7220587

[25] S. Sankararaman, N. Patterson, H. Li, S. Pääbo, D. Reich, The date of interbreeding between Neandertals and modern humans. PLoS Genet. 8, e1002947 (2012). 23055938 10.1371/journal.pgen.1002947 PMC3464203

[26] F. Mafessoni., A high-coverage Neandertal genome from Chagyrskaya Cave. Proc. Natl. Acad. Sci. U.S.A. 117, 15132–15136 (2020). 32546518 10.1073/pnas.2004944117 PMC7334501

[27] K. Prüfer., A high-coverage Neandertal genome from Vindija Cave in Croatia. Science 358, 655–658 (2017). 28982794 10.1126/science.aao1887 PMC6185897

[28] M. Meyer., A high-coverage genome sequence from an archaic Denisovan individual. Science 338, 222–226 (2012). 22936568 10.1126/science.1224344 PMC3617501

[29] E. Huerta-Sánchez., Altitude adaptation in Tibetans caused by introgression of Denisovan-like DNA. Nature 512, 194–197 (2014). 25043035 10.1038/nature13408 PMC4134395

[30] K. E. Langergraber., Generation times in wild chimpanzees and gorillas suggest earlier divergence times in great ape and human evolution. Proc. Natl. Acad. Sci. U.S.A. 109, 15716–15721 (2012). 22891323 10.1073/pnas.1211740109 PMC3465451

[31] A. Kong., A high-resolution recombination map of the human genome. Nat. Genet. 31, 241–247 (2002). 12053178 10.1038/ng917

[32] F. L. Mendez, J. C. Watkins, M. F. Hammer, Neandertal origin of genetic variation at the cluster of OAS immunity genes. Mol. Biol. Evol. 30, 798–801 (2013). 23315957 10.1093/molbev/mst004

[33] A. R. Martin., Human demographic history impacts genetic risk prediction across diverse populations. Am. J. Hum. Genet. 100, 635–649 (2017). 28366442 10.1016/j.ajhg.2017.03.004 PMC5384097

[34] U. Y. Choi, J.-S. Kang, Y. S. Hwang, Y.-J. Kim, Oligoadenylate synthase-like (OASL) proteins: Dual functions and associations with diseases. Exp. Mol. Med. 47, e144 (2015). 25744296 10.1038/emm.2014.110 PMC4351405

[35] A. J. Sams., Adaptively introgressed Neandertal haplotype at the OAS locus functionally impacts innate immune responses in humans. Genome Biol. 17, 246 (2016). 27899133 10.1186/s13059-016-1098-6 PMC5129249

[36] H. Li.; for UK Primary Sjögren’s Syndrome Registry, Identification of a Sjögren’s syndrome susceptibility locus at OAS1 that influences isoform switching, protein expression, and responsiveness to type I interferons. PLoS Genet. 13, e1006820 (2017). 28640813 10.1371/journal.pgen.1006820 PMC5501660

[37] V. Bonnevie-Nielsen., Variation in antiviral 2′,5′-oligoadenylate synthetase (2‘5’AS) enzyme activity is controlled by a single-nucleotide polymorphism at a splice-acceptor site in the OAS1 gene. Am. J. Hum. Genet. 76, 623–633 (2005). 15732009 10.1086/429391 PMC1199299

[38] J. K. Lim., Genetic variation in OAS1 is a risk factor for initial infection with West Nile virus in man. PLoS Pathog. 5, e1000321 (2009). 19247438 10.1371/journal.ppat.1000321 PMC2642680

[39] M. K. El Awady., Single nucleotide polymorphism at exon 7 splice acceptor site of OAS1 gene determines response of hepatitis C virus patients to interferon therapy. J. Gastroenterol. Hepatol. 26, 843–850 (2011). 21182542 10.1111/j.1440-1746.2010.06605.x PMC7166793

[40] J. He., Association of SARS susceptibility with single nucleic acid polymorphisms of OAS1 and MxA genes: A case-control study. BMC Infect. Dis. 6, 106 (2006). 16824203 10.1186/1471-2334-6-106 PMC1550407

[41] M. D. Sørensen., Severe acute respiratory syndrome (SARS): Development of diagnostics and antivirals. Ann. N. Y. Acad. Sci. 1067, 500–505 (2006). 16804033 10.1196/annals.1354.072 PMC7167626

[42] David Reich Lab, Allen Ancient DNA Resource (AADR): Downloadable genotypes of present-day and ancient DNA data, version 42.4, https://reich.hms.harvard.edu/allen-ancient-dna-resource-aadr-downloadable-genotypes-present-day-and-ancient-dna-data. Accessed 19 April 2020.

[43] S. Yair, K. M. Lee, G. Coop, The timing of human adaptation from Neanderthal introgression. bioRxiv, [Preprint] (2020). 2020.10.04.325183. Accessed 30 November 2020. 10.1093/genetics/iyab052 PMC8128397 33787889

[44] F. L. Mendez, J. C. Watkins, M. F. Hammer, Global genetic variation at OAS1 provides evidence of archaic admixture in Melanesian populations. Mol. Biol. Evol. 29, 1513–1520 (2012). 22319157 10.1093/molbev/msr301

[45] C. M. Carey., Recurrent loss-of-function mutations reveal costs to OAS1 antiviral activity in primates. Cell Host Microbe 25, 336–343.e4 (2019). 30713099 10.1016/j.chom.2019.01.001 PMC6609161

[46] A. D. Yates., Ensembl 2020. Nucleic Acids Res. 48, D682–D688 (2020). 31691826 10.1093/nar/gkz966 PMC7145704

