# Supplementary material for: A genomic region associated with protection against severe COVID-19 is inherited from Neandertals
Source: Proc Natl Acad Sci U S A. 2021 Feb 15;118(9):e2026309118. doi: 10.1073/pnas.2026309118 (PMC7936282; doi:10.1073/pnas.2026309118)
Supplement: Supplementary File [file pnas.2026309118.sapp.pdf]

PNAS
www.pnas.org

Supplementary Information for

A genomic region associated with protection against severe COVID-19 is inherited from Neandertals

Hugo Zeberg and Svante Pääbo

Hugo Zeberg and Svante Pääbo
Email: hugo.zeberg@ki.se, paabo@eva.mpg.de

This PDF file includes:

Tables S1 to S2
SI References

Table S1. Variants associated with COVID-19 severity in the OAS genomic region. P-values (<1e-5) from the COVID-19 Host Genetics Imitative, not including data from 23andMe. Analysis include 7,885 hospitalized COVID-19 patients and 961,804 population controls. LD indicate r2 with the index variant of the GenOMICC study (rs10735059)(1) for the entire 1000 Genomes data set (2) and the Eurasian subset thereof, respectively. 'Ref' gives the human reference allele (hg19) and 'Alt' the alternative allele.

Chr	Pos	rsid	P-value	LD (rs10735079)	Eurasian LD (rs10735079)	Ref	Alt
12	113350796	rs2057778	2.1e-06	0.71	0.94	G	T
12	113352159	rs4767023	9.9e-07	0.34	0.93	T	C
12	113357193	rs10774671	2.6e-07	0.27	0.93	G	A
12	113357209	rs1131476	9.8e-08	0.69	0.95	G	A
12	113357442	rs2660	1.0e-07	0.69	0.95	G	A
12	113358106	rs7135577	1.1e-06	0.69	0.95	A	G
12	113358791	rs4767024	6.3e-07	0.71	0.94	T	C
12	113358794	rs4767025	7.9e-07	0.71	0.94	C	T
12	113359132	rs4767026	6.2e-07	0.68	0.95	A	G
12	113359157	rs4767027	7.1e-07	0.72	0.95	T	C
12	113359188	rs4767028	7.5e-07	0.69	0.95	A	G
12	113359318	rs4767029	7.7e-07	0.71	0.94	G	A
12	113359577	rs4767030	6.9e-07	0.69	0.94	C	T
12	113359703	rs10850092	7.9e-07	0.71	0.94	C	G
12	113360025	rs6489864	9.9e-07	0.72	0.95	A	G
12	113360302	rs6489865	9.7e-07	0.72	0.95	A	G
12	113360468	rs10850093	7.6e-07	0.71	0.95	C	T
12	113360563	rs10850094	7.5e-07	0.72	0.95	T	C
12	113360575	rs10850095	7.5e-07	0.72	0.95	T	C
12	113360737	rs10774672	6.0e-07	0.68	0.95	G	T
12	113360786	rs10850096	7.6e-07	0.72	0.95	T	C
12	113361117	rs10850097	5.0e-07	0.47	0.94	C	T
12	113361158	rs10774673	5.8e-07	0.71	0.95	C	T
12	113361174	rs10774674	5.8e-07	0.71	0.95	T	C
12	113361237	rs10774675	5.7e-07	0.71	0.93	C	T
12	113361443	rs11066451	6.3e-07	0.73	0.95	G	A
12	113361574	rs11066452	6.2e-07	0.73	0.96	G	T
12	113362058	rs10850098	6.4e-07	0.75	0.98	G	C
12	113362407	rs10774676	7.9e-07	0.75	0.99	A	G
12	113362421	rs10774677	7.7e-07	0.75	0.99	G	A
12	113362751	rs4767031	1.4e-06	0.75	0.99	C	G
12	113362974	rs4766663	9.4e-07	0.75	0.98	A	G
12	113362997	rs4766664	1.3e-07	0.75	0.99	T	G
12	113363077	rs4767032	6.8e-07	0.75	0.99	T	G
12	113363109	rs4766665	8.1e-07	0.75	0.99	T	C
12	113363178	rs4988618	7.6e-07	0.75	0.99	G	A
12	113363284	rs7958379	7.8e-07	0.75	0.99	A	G
12	113363408	rs6489866	6.3e-07	0.75	0.98	A	G
12	113363550	rs6489867	2.9e-07	0.51	0.98	C	T
12	113363692	rs6489868	7.4e-07	0.75	0.98	G	C
12	113363922	rs6489869	7.5e-07	0.75	0.98	A	C
12	113363972	rs6489870	7.2e-07	0.75	0.98	G	A
12	113364332	rs4766666	7.0e-07	0.75	0.98	A	G
12	113364382	rs4767033	3.6e-07	0.75	0.98	T	A

Table S1. Cont'd from previous page.

Chr	Pos	rsid	P-value	LD (rs10735079)	Eurasian LD (rs10735079)	Ref	Alt
12	113364471	rs4767034	6.9e-07	0.75	0.98	A	G
12	113364504	rs10850099	6.9e-07	0.75	0.98	A	G
12	113364512	rs10850100	7.1e-07	0.75	0.98	T	C
12	113364633	rs4766667	6.1e-07	0.75	0.99	G	A
12	113364780	rs4766668	9.0e-07	0.75	0.98	T	C
12	113365065	rs4766671	7.4e-07	0.75	0.99	A	G
12	113365148	rs4766672	9.4e-07	0.72	0.99	A	G
12	113365161	rs4766673	7.0e-07	0.75	0.99	A	G
12	113365201	rs4766674	7.1e-07	0.75	0.99	G	T
12	113365453	rs4766675	7.3e-07	0.75	0.99	A	T
12	113365581	rs4766676	1.0e-06	0.75	0.99	C	T
12	113365667	rs7304898	7.3e-07	0.75	0.99	T	C
12	113365687	rs7315441	7.0e-07	0.75	0.99	G	T
12	113365803	rs7305035	7.2e-07	0.75	0.99	T	C
12	113365828	rs7316586	7.4e-07	0.75	0.99	C	T
12	113366049	rs916972	7.3e-07	0.75	0.99	G	T
12	113366691	rs7134391	2.8e-07	0.52	0.98	G	A
12	113366899	rs7306205	6.0e-07	0.75	0.99	A	G
12	113367309	rs1859336	6.1e-07	0.75	0.98	C	T
12	113367343	rs2384071	7.4e-07	0.75	0.98	A	G
12	113367422	rs2384072	6.0e-07	0.75	0.99	T	C
12	113367595	rs2384073	6.7e-07	0.75	0.99	A	G
12	113367895	rs6489874	6.9e-07	0.75	0.99	A	G
12	113368030	rs6489875	7.2e-07	0.75	0.98	G	A
12	113368055	rs6489876	5.9e-07	0.75	0.98	G	A
12	113368079	rs6489877	5.8e-07	0.75	0.98	A	G
12	113368125	rs6489878	6.5e-07	0.75	0.99	A	G
12	113368249	rs7298184	6.9e-07	0.75	0.99	C	A
12	113368505	rs12322160	6.5e-07	0.75	0.99	A	G
12	113368605	rs7132404	6.5e-07	0.75	0.99	T	C
12	113369177	rs4767036	7.7e-07	0.74	0.99	T	G
12	113370427	rs1154970	3.0e-07	0.53	0.98	A	C
12	113370966	rs1859333	6.0e-07	0.75	0.99	T	C
12	113371523	rs1981554	8.0e-07	0.75	0.99	G	C
12	113371646	rs9971885	5.9e-07	0.75	0.99	A	C
12	113371973	rs57484342	7.1e-07	0.75	0.99	A	G
12	113371977	rs61266553	7.4e-07	0.75	0.99	A	G
12	113372539	rs7966314	5.0e-07	0.74	0.99	A	G
12	113372804	rs4767037	2.6e-07	0.74	0.99	A	C
12	113372866	rs1981557	2.6e-07	0.74	0.99	G	C
12	113372961	rs1981556	2.9e-07	0.75	0.99	C	G
12	113372977	rs1981555	2.7e-07	0.74	0.99	G	A
12	113373563	rs3759376	3.0e-07	0.76	0.99	A	G
12	113373588	rs3759375	3.1e-07	0.76	0.99	A	G

Table S1. Cont'd from previous page.

Chr	Pos	rsid	P-value	LD (rs10735079)	Eurasian LD (rs10735079)	Ref	Alt
12	113374017	rs4767040	3.7e-07	0.74	0.99	G	C
12	113374748	rs10774679	9.9e-08	0.54	0.98	C	T
12	113375036	rs7132797	3.6e-07	0.74	0.99	A	C
12	113375983	rs1156361	3.7e-07	0.76	0.99	T	C
12	113376320	rs3815178	4.7e-07	0.76	0.99	C	T
12	113376388	rs1859330	1.8e-06	0.36	0.98	G	A
12	113376913	rs7299132	2.5e-07	0.76	1.00	T	A
12	113377822	rs6489879	3.6e-07	0.76	1.00	G	A
12	113378081	rs4238033	3.9e-07	0.76	1.00	T	A
12	113378677	rs4767041	1.5e-06	1.00	1.00	G	A
12	113379039	rs7955267	1.3e-06	1.00	1.00	C	T
12	113379123	rs7311182	3.7e-07	0.76	0.99	T	C
12	113380008	rs10735079	4.4e-07	1.00	1.00	G	A
12	113380271	rs6489880	3.7e-07	0.76	1.00	C	T
12	113380529	rs7980275	3.0e-06	0.98	1.00	T	A
12	113380708	rs7977345	1.5e-06	1.00	1.00	A	T
12	113381217	rs6489881	1.4e-06	1.00	1.00	A	T
12	113381376	rs6489882	3.6e-07	0.75	0.97	G	A
12	113381695	rs7131998	8.2e-08	0.75	0.93	A	C
12	113381749	rs7135260	3.9e-07	0.50	0.91	T	C
12	113381956	rs2269899	3.2e-08	0.50	0.88	C	T
12	113382977	rs2384074	4.0e-07	0.55	0.65	C	T
12	113385000	rs10850103	1.9e-07	0.48	0.56	A	T
12	113386950	rs2285932	1.3e-07	0.48	0.56	T	C
12	113392182	rs7310667	1.0e-07	0.31	0.56	A	G
12	113396010	rs10744789	4.3e-08	0.29	0.56	T	C
12	113397143	rs4238034	4.7e-08	0.29	0.56	T	C
12	113402899	rs4767044	1.2e-07	0.48	0.56	C	A
12	113405181	rs1557866	1.3e-07	0.48	0.56	A	C
12	113406196	rs3937434	1.0e-07	0.48	0.56	A	G
12	113406460	rs2016831	1.1e-07	0.48	0.56	G	C
12	113406945	rs757405	1.1e-07	0.48	0.56	T	A
12	113408208	rs2010604	2.6e-07	0.50	0.56	G	C
12	113409413	rs4767045	1.0e-07	0.48	0.56	C	T
12	113410316	rs10744791	7.1e-08	0.48	0.56	G	A
12	113418850	rs1298962	1.5e-06	0.36	0.49	G	C
12	113419177	rs1293774	1.,e-06	0.36	0.49	C	T
12	113420604	rs1293772	1.4e-06	0.36	0.49	A	G
12	113421295	rs1293771	1.2e-06	0.36	0.49	A	G
12	113424501	rs1293768	1.7e-06	0.36	0.49	T	G
12	113425154	rs1293767	4.1e-07	0.43	0.49	C	G
12	113425493	rs1293765	1.7e-06	0.36	0.49	T	C
12	113425679	rs1293764	1.6e-06	0.36	0.49	T	C

Table S2. Genome-wide significant SNPs (p < 5e-8) on chromosome 12. The p-values are lower than in Fig. 1 due inclusion of data from 23andMe. Red marks the minor allele, which in all cases shown is protective. Note that the human reference genome carries the Neandertal haplotype. *'Ref', 'Alt',* and *'Anc'* refer to the reference, alternative and ancestral allele, respectively. LD denotes linkage disequilibrium in the 1000 Genomes Project dataset (r^2) (2), p-values from release four of the Covid-19 Host Genetics Intiative (3).

Chr	Pos	rsid	P-value	LD (rs4766664)	Ref	Alt	Anc	Vindija	Altai	Chagyrskaya	Denisova
12	113362997	rs4766664	5.7e-09	1.00	T	G	T	T/T	T/T	T/T	G/G
12	113381956	rs2269899	7.9e-09	0.56	C	T	T	C/C	C/C	C/C	T/T
12	113380008	rs10735079	7.9e-09	0.75	G	A	A	G/G	G/G	G/G	A/A
12	113410316	rs10744791	1.3e-08	0.63	G	A	A	G/G	A/G	G/G	A/A
12	113372804	rs4767037	1.4e-08	0.98	A	C	A	A/A	A/A	A/A	C/C
12	113372866	rs1981557	1.4e-08	0.98	G	C	G	G/G	G/G	G/G	C/C
12	113372977	rs1981555	1.4e-08	0.98	G	A	G	.	.	.	.
12	113376913	rs7299132	1.5e-08	0.99	T	A	A	T/T	T/T	T/T	A/A
12	113357209	rs1131476	1.6e-08	0.92	G	A	G	G/G	G/G	G/G	A/A
12	113357442	rs2660	1.6e-08	0.92	G	A	G	G/G	G/G	G/G	A/A
12	113381695	rs7131998	1.8e-08	0.86	A	C	A	.	.	.	.
12	113406196	rs3937434	1.8e-08	0.63	A	G	G	.	.	.	.
12	113406460	rs2016831	1.8e-08	0.63	G	C	C	.	.	.	.
12	113374017	rs4767040	1.9e-08	0.98	G	C	G	G/G	G/G	G/G	C/C
12	113372961	rs1981556	1.9e-08	1.00	C	G	G	.	.	.	.
12	113406945	rs757405	1.9e-08	0.63	T	A	A	T/T	A/T	T/T	A/A
12	113373563	rs3759376	1.9e-08	1.00	A	G	G	A/A	A/A	A/A	G/G
12	113409413	rs4767045	1.9e-08	0.63	C	T	T	T/T	T/T	C/C	T/T
12	113402899	rs4767044	2.0e-08	0.63	C	A	A	C/C	C/C	C/C	A/A
12	113373588	rs3759375	2.0e-08	1.00	A	G	G	A/A	A/A	A/A	G/G
12	113386950	rs2285932	2.0e-08	0.63	T	C	C	T/T	T/T	T/T	C/C
12	113375036	rs7132797	2.1e-08	0.98	A	C	A	A/A	A/A	A/A	C/C
12	113392182	rs7310667	2.1e-08	0.31	A	G	A	.	.	.	.
12	113381376	rs6489882	2.2e-08	0.97	G	A	A	A/A	A/A	A/A	A/A
12	113377822	rs6489879	2.3e-08	0.99	G	A	G	G/G	G/G	G/G	A/A
12	113379123	rs7311182	2.3e-08	0.99	T	C	C	.	.	.	.
12	113405181	rs1557866	2.4e-08	0.63	A	C	C	A/A	A/A	A/A	C/C
12	113380271	rs6489880	2.4e-08	0.99	C	T	T	.	.	.	.
12	113364382	rs4767033	2.4e-08	1.00	T	A	A	T/T	T/T	T/T	A/A
12	113378081	rs4238033	2.4e-08	0.99	T	A	A	T/T	T/T	T/T	A/A
12	113375983	rs1156361	2.6e-08	1.00	T	C	C	T/T	T/T	T/T	C/C
12	113372539	rs7966314	3.1e-08	0.98	A	G	A	A/A	A/A	A/A	G/G
12	113408208	rs2010604	3.2e-08	0.52	G	C	C	G/G	G/G	G/G	G/G
12	113374748	rs10774679	3.3e-08	0.44	C	T	C	C/C	C/C	C/C	C/C
12	113385000	rs10850103	3.3e-08	0.63	A	T	T	A/A	T/A	A/A	T/T
12	113376320	rs3815178	3.3e-08	0.99	C	T	C	C/C	C/C	C/C	T/T
12	113376388	rs1859330	3.4e-08	0.53	G	A	G	G/G	G/G	G/G	A/A
12	113381217	rs6489881	3.6e-08	0.75	A	T	A	A/A	A/A	A/A	T/T
12	113380708	rs7977345	3.7e-08	0.75	A	T	T	A/A	A/A	A/A	T/T
12	113379039	rs7955267	3.7e-08	0.75	C	T	C	.	.	.	.
12	113382977	rs2384074	3.7e-08	0.66	C	T	C	.	.	.	.
12	113396010	rs10744789	4.1e-08	0.35	T	C	C	.	.	.	.
12	113371646	rs9971885	4.2e-08	1.00	A	C	C	.	.	.	.
12	113378677	rs4767041	4.3e-08	0.75	G	A	G	.	.	.	.
12	113367422	rs2384072	4.6e-08	1.00	T	C	T	T/T	T/T	T/T	C/C
12	113366899	rs7306205	4.6e-08	1.00	A	G	A	A/A	A/A	A/A	G/G
12	113367309	rs1859336	4.7e-08	1.00	C	T	C	C/C	C/C	C/C	T/T
12	113364633	rs4766667	4.8e-08	1.00	G	A	G	.	.	.	.
12	113370966	rs1859333	4.9e-08	1.00	T	C	C	T/T	T/T	T/T	C/C
12	113368079	rs6489877	4.9e-08	0.99	A	G	A	.	.	.	.
12	113363408	rs6489866	4.9e-08	1.00	A	G	A	.	.	.	.

References

1. E. Pairo-Castineira, et al., Genetic mechanisms of critical illness in Covid-19. Nature (2020) https:/doi.org/10.1038/s41586-020-03065-y.
2. A. Auton, et al., A global reference for human genetic variation. Nature 526, 68–74 (2015).
3. COVID-19 Host Genetics Intitative, The COVID-19 Host Genetics Initiative, a global initiative to elucidate the role of host genetic factors in susceptibility and severity of the SARS-CoV-2 virus pandemic. Eur J Hum Genet 28, 715–718 (2020).
